# Supplementary material for: The Efficacy of Re-Warm-Up Practices during Half-Time: A Systematic Review
Source: Medicina (Kaunas). 2021 Sep 17;57(9):976. doi: 10.3390/medicina57090976 (PMC8466564; doi:10.3390/medicina57090976)
Supplement: Supplementary file 1 [file medicina-57-00976-s001.zip › medicina-1336630-supplementary.pdf]

Electronic Supplementary Material Table S1. Full search strategy for each database with arguments presented as they were used.

Data Base	Search strategy	Results
Web Of Science	(((TS=(re-warm-up)) OR TS= (half-time strategy)) OR TS= (["second-half" AND "warm-up"])) OR TS= (RW-U strategy)	325
Scopus	re-warm-up OR half-time AND strategy OR ["second-half" AND "warm-up"] OR RW-U strategy	213
SportDiscus	re-warm-up OR RW-U strategy OR (half-time AND strategy) OR (second-half AND warm-up)	62
PubMed	(re-warm-up) OR (half-time AND strategy) OR (second-half AND warm-up) OR (RWU strategy)	158
	**TOTAL**	758
